# DIALysis or not: Outcomes in older kidney patients with GerIatriC Assessment (DIALOGICA): rationale and design

**DOI:** 10.1186/s12882-021-02235-y

**Published:** 2021-01-23

**Authors:** Mathijs van Oevelen, Alferso C. Abrahams, Willem Jan W. Bos, Mariëlle H. Emmelot-Vonk, Simon P. Mooijaart, Merel van Diepen, Brigit C. van Jaarsveld, Anita van Eck van der Sluijs, Carlijn G. N. Voorend, Marjolijn van Buren, on behalf of the DIALOGICA study group, J. B. van der Net, T. T. Cnossen, K. Goossens, H. P. Krepel, S. J. J. Logtenberg, C. R. Susanto, D. Severs, H. A. Polinder-Bos, M. A. Siezenga, R. Bogers, E. K. Hoogeveen, A. P. M. Kerckhoffs, T. Cornelis, J. T. Jonker, J. M. H. Joosten, E. J. R. Litjens, A. Y. Adema, A. Bontemps-Visser, R. J. Bosma, M. D. M. Romijn, H. Boom, G. van Kempen, H. H. T. I. Klein, J. D. Snoep, M. H. P. J. Schuurmans, F. L. Nauta, C. F. M. Franssen, F. M. Molenaar, I. Wauters

**Affiliations:** 1 Department of Internal Medicine, Leiden University Medical Center, Albinusdreef 2, 2333 ZA Leiden, The Netherlands; 2 Department of Nephrology and Hypertension, University Medical Center Utrecht, Utrecht, The Netherlands; 3 Department of Internal Medicine, St Antonius Hospital, Nieuwegein, The Netherlands; 4 Department of Geriatrics, University Medical Center Utrecht, Utrecht, The Netherlands; 5 Department of Gerontology and Geriatrics, Leiden University Medical Center, Leiden, The Netherlands; 6 Department of Clinical Epidemiology, Leiden University Medical Center, Leiden, The Netherlands; 7 Department of Nephrology, Amsterdam Cardiovascular Sciences, Amsterdam University Medical Center, Amsterdam, The Netherlands; 8 Department of Nephrology, Haga Hospital, The Hague, The Netherlands

**Keywords:** Costs and cost analysis, Dialysis, Frailty, geriatric assessment, Kidney failure, chronic, Mortality, Quality of life

## Abstract

**Background:**

The incidence and prevalence of older patients with kidney failure who are dependent on dialysis is increasing. However, observational studies showed limited or no benefit of dialysis on mortality in subgroups of these patients when compared to conservative care. As the focus is shifting towards health-related quality of life (HRQoL), current evidence of effects of conservative care or dialysis on HRQoL in older patients is both limited and biased. Dialysis comes with both high treatment burden for patients and high costs for society; better identification of patients who might not benefit from dialysis could result in significant cost savings. The aim of this prospective study is to compare HRQoL, clinical outcomes, and costs between conservative care and dialysis in older patients.

**Methods:**

The *DIALysis or not: Outcomes in older kidney patients with GerIatriC Assessment* (DIALOGICA) study is a prospective, observational cohort study that started in February 2020. It aims to include 1500 patients from 25 Dutch and Belgian centres. Patients aged ≥70 years with an eGFR of 10–15 mL/min/1.73m^2^ are enrolled in the first stage of the study. When dialysis is initiated or eGFR drops to 10 mL/min/1.73m^2^ or lower, the second stage of the study commences. In both stages nephrogeriatric assessments will be performed annually, consisting of questionnaires and tests to assess most common geriatric domains, i.e. functional, psychological, somatic, and social status. The primary outcome is HRQoL, measured with the Twelve-item Short-Form Health Survey. Secondary outcomes are clinical outcomes (mortality, hospitalisation, functional status, cognitive functioning, frailty), cost-effectiveness, and decisional regret. All outcomes are (repeated) measures during the first year of the second stage. The total follow-up will be a maximum of 4 years with a minimum of 1 year in the second stage.

**Discussion:**

By generating more insight in the effects of conservative care and dialysis on HRQoL, clinical outcomes, and costs, findings of this study will help patients and physicians make a shared decision on the best individual treatment option for kidney failure.

**Trial registration:**

The study was registered in the Netherlands Trial Register (NL-8352) on 5 February 2020.

**Supplementary Information:**

The online version contains supplementary material available at 10.1186/s12882-021-02235-y.

## Background

In the Netherlands, over 6200 patients (358 per million population) with kidney failure are currently dependent on dialysis with an annual incidence of over 1650 patients (95 per million population) [1]. The proportion of older patients has risen over the years and in 2019 45% of the incident dialysis patients were 70 years or older [2]. After dialysis initiation, the overall mortality in this age group is high: 18% in the first year, 72% after 5 years [2]. A Dutch retrospective single-centre study showed no survival benefit from dialysis initiation for patients aged 80 years or older and in those above 70 years with severe comorbidity, compared to conservative care (CC) [3]. These results were confirmed by a prospective multicentre study performed in the Netherlands [4]. Results such as these have shifted the focus from mortality towards health-related quality of life (HRQoL) as the most important outcome [5]. Dialysis is associated with a high treatment burden in older patients when compared to CC [6, 7]. A recent meta-analysis reviewed 11 studies comparing HRQoL between older patients treated with CC or dialysis [7]. All studies had a small sample size, eight were single-centre, and eight were highly susceptible to selection bias and/or confounding. Thus, current evidence for the effects of CC or dialysis on HRQoL is both limited and biased. Despite these limitations, the authors conclude that CC has the potential to achieve similar HRQoL as dialysis.

Outcomes such as survival and HRQoL might differ greatly among older patients with kidney failure due to substantial heterogeneity with respect to functional and cognitive impairment and frailty. Establishing which of these conditions are associated with poor outcome may help to identify those patients at highest risk and thus guide informed treatment decisions [8]. To do this, some authors suggest tailoring the comprehensive geriatric assessments used in geriatric care to routine kidney failure care [9]. However, studies showing the benefit of these assessments on outcomes, such as survival and HRQoL, in these patients are lacking.

Besides the impact on survival and HRQoL, dialysis is also a highly expensive treatment: the costs in the Netherlands approximate € 92,000 per patient per year, making it among the most expensive treatment in internal medicine, both per individual patient as in total treatment costs [10]. If dialysis treatment appears to yield little or no benefit in selected older and/or frail patients, such as improved survival or HRQoL, CC could be considered more often as the preferred treatment option. This may result in significant cost savings for treatment of kidney failure.

Therefore, large and well-designed, prospective, multicentre studies with sufficient follow-up in older patients treated with CC or dialysis are needed. The *DIALysis or not: Outcomes in older kidney patients with GerIatriC Assessment* (DIALOGICA) study aims to compare HRQoL, clinical outcomes, and financial costs between CC and dialysis in patients of 70 years or older with kidney failure. Secondly, the study aims to associate clinical and geriatric patient characteristics in the decision-making trajectory with these outcomes. Our hypothesis is that CC is associated with comparable HRQoL, comparable clinical outcomes, and lower costs, compared to dialysis in subgroups of older patients with kidney failure.

## Methods

### Study design

DIALOGICA is a multicentre, prospective, observational cohort study comparing CC with dialysis. The first patient was recruited in February 2020. The study aims to include 1500 patients from at least 25 academic and non-academic centres in the Netherlands and Belgium. DIALOGICA is one of the *Leading the Change* healthcare evaluation projects. *Leading the Change* finances and guides several healthcare evaluation projects in the Netherlands. The programme is financed by Dutch health insurers and aims to increase and implement acquired knowledge from these projects, thereby increasing the effectiveness of the Dutch healthcare system.

**Fig. 1:**
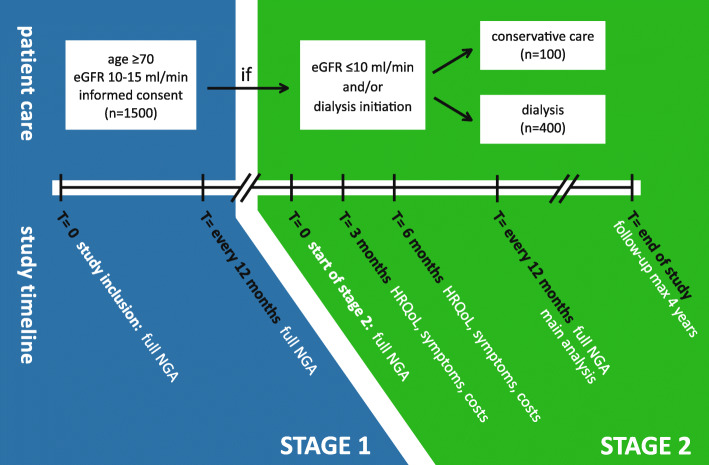
Study flow chart. Abbreviations: *eGFR* estimated Glomerular Filtration Rate, *HRQoL* Health-Related Quality of Life, *NGA* NephroGeriatric Assessment

The study consists of two stages (Fig. 1). Patients remain in the first stage as long as their estimated glomerular filtration rate (eGFR), as calculated by the Chronic Kidney Disease Epidemiology Collaboration (CKD-EPI) formula or using mean urea and creatinine clearances in 24-h urine collections, stays above 10 mL/min/1.73m^2^. The second stage commences at the start of dialysis or when the eGFR drops to or below 10 mL/min/1.73m^2^. This creates two groups (patients treated with either dialysis or CC) which are used as the main determinant for the study.

DIALOGICA is aimed to run for a total of 4 years with a minimum follow-up of 1 year in the second stage. The study is conducted according to the principles of the Declaration of Helsinki and the ICH-GCP guidelines. Primary ethical approval was obtained from the medical research ethics committee of the The Hague region (METC Zuidwest Holland) on May 24th 2019 (reference number 19–071). Local medical research ethics committees in participating centres will also be asked for local ethical approval. The study is registered in the Netherlands Trial Register under NL-8352.

### Study population

Inclusion criteria are age of 70 years and older and an eGFR between 10 and 15 mL/min/1.73m^2^ at the moment of inclusion. Due to the nature of the assessments that will be performed in this study (described in detail later), illiterate patients and those unable to speak Dutch will be excluded. All patients have to provide written informed consent. When a patient appears to have severe cognitive impairment, a geriatrician will judge if a patient is mentally competent to provide the required consent.

### Data registration

At baseline, all relevant demographical and clinical data will be collected. In the first stage of the study, a follow-up visit will be scheduled once-yearly until the start of the second stage. In the second stage, data collection will be performed at start, after 3 months, 6 months, 12 months, and thereafter annually until end of follow-up or end of the study. All data will be recorded in a database developed in collaboration with Nefrovisie. Nefrovisie is the Dutch Quality Institute for Nephrology which hosts the Renine-database, in which all Dutch patients on kidney replacement therapy are registered. Upon consent, data for DIALOGICA will be linked to the Renine-database and the databases of three Dutch studies with similar or complementary study aims and outcomes: the *Dutch nOcturnal and hoME dialysis Study To Improve Clinical Outcomes* (DOMESTICO) study will compare HRQoL, clinical outcomes and costs in home dialysis patients with in-centre haemodialysis patients; the *Pathway for OLDer patients with End-stage Renal disease* (POLDER) project will assess the feasibility of implementing a geriatric assessment in routine predialysis care for older patients with an eGFR < 20 mL/min/1.73m^2^ and the *Optimizing Access Surgery In Senior haemodialysis patients* (OASIS) study will compare three strategies of vascular access in older patients using a randomised design [11–13]. The DIALOGICA database will be archived for future research during a minimum of 15 years after the completion of this study.

### Nephrogeriatric assessment

To assess the most common geriatric domains (i.e. functional, psychological, somatic, and social status) and the study’s outcome measures (i.e. HRQoL, clinical outcomes, and costs), a geriatric assessment tailored to the kidney failure population will be used. It is derived from the consensus-based test set used in a pilot study [11]. This nephrogeriatric assessment (NGA) consists of a combination of 12 questionnaires and seven tests and assessments (Table 1). Nine questionnaires are answered independently by the patient at home and three are conducted by an interviewer. All of the tests and assessments are performed in the participating centres. The NGA is performed at baseline and at a yearly interval during the first stage. Upon entering the second stage, a new NGA will be performed, repeated again at yearly intervals. The questionnaires on HRQoL, symptoms and costs are also repeated after 3 and 6 months into the second stage. To minimise inter-observer bias, the tests will be performed by trained geriatric or (pre) dialysis nurses only.

**Table 1:** The nephrogeriatric assessment used in DIALOGICA

Domain	Measure	Type	Explanation	Scale / cut-off
Functional status	Activities of Daily Living (Katz) [14]	SAQ	Grading of dependency on 6 functions, e.g. bathing, dressing.	0–12, higher scores indicating more dependency, no cut-off values.
	Instrumental Activities of Daily Living (Lawton) [15]	SAQ	Grading of dependency on 8 more complex functions, e.g. handling finances, driving.	0–8 for women, 0–5 for men. Higher scores indicating more independency, no cut-off values.
	Fall risk assessment	IAQ	One-year history of falling and graded fear of falling.	Yes/no; 1 (‘no fear’) to 10 (‘very afraid’).
	Hand grip strength	T	Measured using a Jamar handheld dynamometer, 3 repetitive measurements for both hands.	Highest measurement is used for analysis. Individual cut-off values based on age and gender [16].
Psychological status	Montreal Cognitive Assessment (MoCA) [17]	T	Screening tool for cognitive impairment in 8 domains (visuospatial, naming, memory, attention, language, abstraction, delayed recall, orientation).	0–30, higher scores indicating better cognitive performance, < 26 indication of cognitive impairment.
	Six-item Cognitive Impairment Test (6-CIT) [18]	T	Fast, 6-item screening tool for dementia, assessing orientation, attention and memory.	0–28, higher scores indicating worse cognitive performance, > 10 indication of cognitive impairment.
	Letter-Digit Substitution Test (LDST) [19]	T	Speed-dependent task to measure speed of processing by matching letters to corresponding numbers provided in the key.	0–135 correct matches after 60 s, reference values based on age, gender and level of education [20].
	Geriatric Depression Scale (GDS-15) [21]	IAQ	Initial two-item screening tool (‘Whooley-questions’) on mood and anhedonia, if any of the 2 positive followed by 15 additional binary questions [22].	Yes/no, 0–15, higher scores indicating more or stronger degree of depressive symptoms, > 5 indicative of depression.
	Life Orientation Test - Revised (LOT-R) [23]	SAQ	10 Items including 4 filler items, 3 items for optimism and 3 items for pessimism. Scored using 5-point Likert scales.	0–12 per construct (optimism or pessimism) or 0–24 for total score (higher scores then indicating more optimism). Reference values based on age and gender [24].
Somatic status	Clinical Frailty Scale [25]	CA	Clinicians judgement of frailty based on 9 pictures with accompanying text.	1 (‘very fit’) to 9 (‘terminally ill’).
	Charlson Comorbidity Index [26]	CA	Comorbid conditions with weighted scores for the condition.	1–6 per condition, total range of 0–33. Higher scores indicating more or more severe comorbidity. No cut-off values.
	Surprise question	CA	Clinicians response to the question: “*Would I be surprised if the patient died in the next 12 months?*”, assessed by either nephrologist or (predialysis) nurse	Yes/no.
	Mini Nutritional Assessment - Short Form (MNA-SF) [27]	IAQ	6 Questions for assessing the risk of malnourishment.	0–7 ‘malnourished’, 8–11 ‘risk of malnourishment’, 12–14 ‘well-nourished’.
HRQoL and symptoms	Twelve-item Short Form Health Survey (SF-12) [28]	SAQ	12 Questions regarding HRQoL, resulting in a mental and physical component score. Scored using 3- and 5-point Likert scales. Used in Dutch nephrological care as PRO.	0–100, higher scores indicating better HRQoL, no cut-off values.
	Dialysis Symptom Index (DSI) [29]	SAQ	30 Questions regarding presence of specific symptoms, when present scored on a 5-point Likert scale. Used in Dutch nephrological care as PRO.	Yes/no, if yes 1 (‘not at all’) to 5 (‘very much’). Total score 0–30 for each ‘yes’, higher scores indicating higher number of symptoms. No cut-off values.
Costs	Five-level EQ-5D (EQ-5D-5L) [30]	SAQ	5 Questions regarding health aspects (mobility, self-care, usual activities, pain/discomfort, anxiety/depression) using a 5-point Likert scale and 1 overall health score on a visual-analogue scale. Used to calculate QALYs in cost-effectiveness analysis	1 (‘no problems’) to 5 (‘unable to/extreme problems’); 0 ‘worst health you can imagine’ to 100 (‘the best health you can imagine’ for the visual-analogue scale. Reference values using Dutch value set [31].
	(subset of questions from) iMTA Productivity & Medical Costs Questionnaire [32, 33]	SAQ	8 General questions and 22 questions on healthcare use in the previous 3 months. Used to calculate healthcare-related costs in cost-effectiveness analysis.	NA
Decisional regret	Treatment Choice Questionnaire & Decisional Conflict Scale (DCS) [34, 35]	SAQ	Questionnaire filled in when treatment choice has been made. Contains 23 questions regarding the choice made.	NA for Treatment Choice Questionnaire; 0–100 for DCS, higher scores indicating higher decisional conflict, no cut-off values.
	Decision Regret Scale (DRS) [36]	SAQ	5 Questions on regret regarding the choice for either conservative care or dialysis on a 5-point Likert scale.	0–25, higher scores indicating stronger feelings of regret. No cut-off or reference values [37].

*Abbreviations*: *CA* Clinician Assessment, *HRQoL* Health-Related Quality of Life, *IAQ* Interviewer-Administered Questionnaire, *iMTA* Institute for Medical Technology Assessment, *PRO* Patient Reported Outcome, *SAQ* Self-Administered Questionnaire, *T* Test, *NA* Not Applicable, *QALY* Quality Adjusted Life Year

#### Functional status

Functionality will be assessed by using four instruments. Firstly, the Activity of Daily Living Scale by Katz et al. assesses the patient’s ability to perform activities of daily living independently [14]. It ranks adequacy of performance regarding the six functions of bathing, dressing, toileting, transferring, continence, and feeding. Secondly, the Instrumental Activities of Daily Living Scale by Lawton et al. measures eight domains of more complex independent living skills, such as handling finances, medication, and driving [15]. Thirdly, hand grip strength will be measured using a Jamar handheld dynamometer. Lastly, patients’ fall risk will be assessed by both asking for fall frequency in the last year and their fear of falling, using a numeric rating scale.

#### Psychological status

Psychological status is assessed by using a total of five instruments, including three cognitive tests. The Montreal Cognitive Assessment scores different types of cognitive abilities, such as orientation, short-term memory, executive function, and visuospatial abilities [17]. The Six-item Cognitive Impairment Test is a fast and simple tool to screen for dementia, consisting of only six questions [18]. Finally, the Letter Digit Subtraction Test is a speed-dependent task that measures complex neuropsychological processes including visual scanning, mental flexibilities, sustained attention, psychomotor speed, and information processing [19].

Depression is assessed in two steps: first two case-finding questions (‘Whooley-questions’) are used, extended with the fifteen-item Geriatric Depression Scale if one or both of the two initial questions are positive [21, 22]. The Life Orientation Test - Revised is a ten-item measure of optimism versus pessimism [23, 24, 38].

#### Somatic status

Somatic status will be evaluated by using three instruments. Treating clinicians will use the Clinical Frailty Score, which grades patients from 1 (‘very fit’) to 9 (‘terminally ill’) [25]. Comorbidity will be assessed with the Charlson Comorbidity Index [26]. Finally, the surprise question, a screening tool in which caregivers answer the question “*Would you be surprised if this patient died within the next twelve months?*”, will be applied [39]. The Mini Nutritional Assessment - Short Form is used to assess the nutritional status of patients [27]. It is a well validated screening tool for the geriatric population consisting of 18 questions, categorising patients as ‘well-nourished’, ‘at risk of malnutrition’ or ‘malnourished’.

#### Social status

Relevant social data (e.g. marital and residential status, biographic data such as educational attainment, and support by home care professionals and/or caregivers) is registered.

#### HRQoL and symptoms

Patients’ HRQoL will be measured by the Twelve-item Short Form Health Survey (SF-12) [28]. Together with the Dialysis Symptom Index (DSI), it was selected as the patient reported outcome measures in Dutch nephrological care by the Dutch Kidney Patients Association, the Dutch Federation for Nephrology, Nefrovisie, and Leiden University Medical Center [29, 40]. The SF-12 and DSI were introduced for all Dutch patients on maintenance dialysis in 2018 [40]. The SF-12 consists of 12 questions regarding HRQoL and is the shorter version of the Short Form-36 (SF-36), the most widely used survey to assess HRQoL [28]. The SF-36 consists of eight domains: physical functioning, role-physical, bodily pain, general health, vitality, social function, role-emotional, and mental health. These domains are summarised in the Physical Component Summary Score and the Mental Component Summary Score. In the SF-12 these summary scores are calculated from the twelve most important items (explaining 90% variance) of the SF-36 questionnaire [41]. As the average difference in summary scores between SF-12 and SF-36 is quite small, for time-efficiency reasons, the SF-12 can be used reliably in cohort studies [42]. The DSI is a set of 30 questions evaluating the severity of symptoms in patients with kidney failure (Table S1) [29]. Patients report the level of burden of the symptoms on a five-point Likert scale, ranging from ‘not at all bothersome’ to ‘very bothersome’. Since these symptoms, such as nausea, energy loss, and shortness of breath, are common in patients with kidney failure and not specifically related to dialysis treatment, this questionnaire is also applicable to patients treated with CC.

#### Costs

For cost-effectiveness analyses, two questionnaires are used to investigate healthcare resources utilisation and patient costs. The Five-level EQ-5D (EQ-5D-5L) is a short and widely used questionnaire in both clinical and health-economic research as its scores can easily be translated into quality adjusted life years (QALYs), the primary outcome measure for most cost-effectiveness research [30, 31]. It consists of five questions on domains of HRQoL, such as mobility, pain, and daily functioning. In addition, patients’ and healthcare costs and costs with regard to productivity losses will be assessed with a subset of questions from the Institute for Medical Technology Assessment (iMTA) Productivity Cost Questionnaire (iPCQ) and the iMTA Medical Cost Questionnaire (iMCQ) [32, 33].

#### Decisional regret

Decisional regret regarding the choice for CC or dialysis, will be determined with the validated Decisional Regret Scale [36]. Multiple studies assessed the regret of patients to start dialysis with results ranging from 7.4% in a Dutch survey up to 61% in a Canadian study [43–46]. Currently, there is a lack of data regarding the decisional regret comparing CC with dialysis. An additional questionnaire (Treatment Choice & Decisional Conflict Scale) regarding the treatment choice made will be added to gain more insight in the decision-making process regarding the choice between CC and dialysis [34, 35].

### Outcome variables

The primary outcome parameter is HRQoL (Table 2). DIALOGICA will assess three secondary outcome parameters: clinical outcomes, cost-effectiveness, and decisional regret. The clinical outcomes that will be assessed are mortality, hospitalisation, functional status, cognitive functioning, and frailty. All outcomes are assessed with (repeated) measures during the first year of the second stage.

**Table 2:** Study outcomes

Outcomes			Instruments
Primary outcome	HRQoL		SF-12 physical and mental component scores [28]
Secondary outcomes	Clinical outcomes	Mortality (all-cause)	ERA-EDTA codes [16, 47]
		Hospitalisation (all-cause)	ICD-10 codes [48]
		Functional status	Katz-ADL [14], Lawton-iADL [15]
		Cognitive functioning	MoCA [17], 6-CIT [18], LDST [20]
		Frailty	Clinical Frailty Scale [25]
	Cost-effectiveness		ICERs, calculated using EQ-5D-5 L [30] and iPCQ/iMCQ [32, 33]
	Decisional regret		Decisional Regret Scale [36]

All outcomes are repeated measures during the first year of the second stage (Fig. 1)

*Abbreviations*: *6-CIT* six-item Cognitive Impairment Test, *EQ-5D-5L* Five-level EQ-5D, *ERA-EDTA* European Renal Association - European Dialysis & Transplant Organisation, *HRQoL* Health-Related Quality of Life, *(i) ADL* (Instrumental) Activities of Daily Living, *ICD-10* International Statistical Classification of Diseases, 10th edition, *ICER* Incremental Cost-Effectiveness Ratio, *iMCQ* iMTA Medical Costs Questionnaire, *iPCQ* iMTA Productivity Cost Questionnaire, *LDST* Letter-Digit Substitution Test, *MoCA* Montreal Cognitive Assessment, *QALY* Quality Adjusted Life Year, *SF-12* Twelve-item Short Form Health Survey

#### HRQoL

HRQoL is assessed by comparing repeated measurements of both the calculated Physical Component Summary Score and the Mental Component Summary Score of the SF-12 at the start of the second stage and after 3, 6 and 12 months after the start of the second stage.

#### Clinical outcomes

Mortality will be analysed as all-cause mortality within the first year of the second stage. The cause of death will also be categorised into seven categories, based on the United Kingdom Renal Registry, using ERA-EDTA codes (Table S2) [16, 47]. Hospitalisation will be analysed as all-cause hospitalisation within the first year of the second stage and each individual episode will be categorised into seven categories, using ICD-10 codes (Table S3) [48]. Functional status, cognitive functioning, and frailty will be repeated measures (at the start of the second stage and after 12 months) using their respective instruments, described in the previous paragraphs.

#### Cost-effectiveness

QALYs will be calculated using the EQ-5D-5L [30]. Total healthcare costs will be calculated using a subset of questions from the iPCQ and iMCQ [32, 33]. Cost-effectiveness will be assessed as total costs per QALY using incremental cost-effectiveness ratio’s (ICERs).

#### Decisional regret

Decisional regret will be measured using the Decisional Regret Scale, measured at 12 months after the start of the second stage only [36].

### Statistical analysis

All statistical analyses will be performed using statistical software, such as SPSS and R. Univariable and multivariable regression analysis to correct for possible confounders will be used to compare groups. Longitudinal data will be analysed with linear and logistic mixed models and presented as estimated coefficients and odds ratios with 95% confidence intervals. Cumulative incidence of hospitalisation and mortality will be reported in Kaplan Meier curves. A Cox proportional hazards model will be used to compare the rate of mortality and hospitalisation between groups. Overall costs and ICERs will be compared across the two groups using linear regression. Discounting, a mathematical procedure for adjusting future costs to their ‘present day value’, will be applied, as requested for all health economic evaluation exceeding a time frame of 1 year. To deal with missing data, multiple imputation by fully conditional specification will be applied.

### Sample size calculation

For the primary outcome, we aim to have 80% power to detect a difference of 3.0 points in the SF-12 summary scores 12 months after the start of the second stage. To reject the null hypothesis of equal means with a standard deviation for both groups of 9.0 with a significance level (alpha) of 0.05 using a two-sided two-sample equal-variance Z-test, a required total sample size of 443 patients was calculated (89 patients on CC, 354 on dialysis, 1:4 ratio based on unpublished data from three Dutch centres). When taking into account a dropout rate of 10%, 99 patients on CC and 394 on dialysis are needed, a total of 493 patients. Based on a study from the United States, we expected one third of patients to progress from stage one to two within 3 years, so a cohort of 1500 patients will be needed [49].

## Discussion

With an ageing population with kidney failure, more patients will face the decisional moment whether to start dialysis or not. As the survival benefit of dialysis treatment in the geriatric population is debatable, focus has shifted towards HRQoL as primary outcome measure. The impact of dialysis on HRQoL of older patients is probably large, but current data comparing it to CC are both limited and biased, creating an urgent need for high-quality studies [7]. DIALOGICA will be the first large study to prospectively assess the differences between patients choosing CC or dialysis. With a total follow-up of up to 4 years, outcome parameters can be assessed in more detail. Biological age, which also takes factors such as functional status, cognitive functioning, and somatic functioning into account, might influence these outcomes more than calendar age. This study will help to determine individual patient characteristics to identify patients less likely to benefit from dialysis initiation. This will support patients with kidney failure and their nephrologists in making a well-informed and shared decision when discussing renal replacement therapy. To do so, DIALOGICA combines a practical test set of well-validated questionnaires and assessments that can be performed in less than 1 hour per patient. With better identification of patients who might not benefit from dialysis, a significant reduction in costs for treatment of kidney failure can be achieved if these patients choose for CC, since CC has far lower annual treatment costs compared to dialysis [6].

DIALOGICA has an observational study design instead of a randomised controlled design. We deemed the patient’s choice between CC and dialysis too fundamentally different to let it be determined by fate. Randomised studies for treatment modalities have been shown to be challenging in patients with kidney failure in general as they have large and different implications for patients’ daily life. For example, a previous Dutch study that tried to randomise patients between haemodialysis and peritoneal dialysis failed and the choice whether or not to start dialysis arguably has even more impact [50]. To our knowledge, only one study comparing CC and dialysis is currently recruiting patients using a randomised design [51]. The implication of the observational design of DIALOGICA is that it is more sensitive to confounding by indication, since observed and unobserved variables can influence outcome of treatment. However, selective inclusion into randomised controlled studies can also lead to poor external validity of their results and an observational study may provide a better reflection of daily clinical practice. To account for the influence of the observed variables on treatment outcome and thus to limit confounding by indication, extensive correction for confounding will be applied, using a large set of patients’ characteristics.

Treating physicians and patients are not blinded for the results of the NGA, because this could hamper appropriate care for newly diagnosed geriatric impairments. Moreover, as NGAs are becoming standard of care in most Dutch centres, it is ethically undesirable to withhold the results of the NGA from treating physicians and participating patients. Since the results of the NGA might influence the treatment decision both for patients and for treating physicians this can introduce selection bias between patients choosing for CC and dialysis, and could be considered a limitation. Where needed, correction for baseline NGA discrepancies will be applied.

## Conclusion

Data on relevant outcomes are needed to answer the question whether CC is a serious alternative to dialysis in (a subgroup of) older patients with kidney failure. In the upcoming years, DIALOGICA will investigate the effect of CC on HRQoL, clinical outcomes, and cost-effectiveness in comparison to dialysis in this population, generating more insight to aid doctors and patients in the shared decision making process.

## Supplementary Information

**Additional file 1: Table S1.** The Dialysis Symptom Index.

**Additional file 2: Table S2.** Categories for mortality, using ERA-EDTA codes, based on the United Kingdom Renal Registry.

**Additional file 3: Table S3.** Categories for hospitalisation, using ICD-10 codes.

## Data Availability

Not applicable.

## Abbreviations

CC: Conservative Care
CKD-EPI: Chronic Kidney Disease EPIdemiology Collaboration
DIALOGICA: DIALysis or not - Outcomes in older kidney patients with GerIatriC Assessment
DSI: Dialysis Symptom Index
eGFR: Estimated Glomerular Filtration Rate
EQ-5D-5L: Five-level EQ-5D questionnaire
HRQoL: Health-Related Quality of Life
ICER: Incremental Cost-Effectiveness Ratio
iMCQ: iMTA Medical Consumption Questionnaire
iMTA: Institute for Medical Technology Assessment
iPCQ: iMTA Productivity Cost Questionnaire
NGA: NephroGeriatric Assessment
SF-12/− 36: 12/36-Item Short Form Health Survey
QALY: Quality adjusted life year
