# Correction to: DIALysis or not: Outcomes in older kidney patients with GerIatriC Assessment (DIALOGICA): rationale and design

**DOI:** 10.1186/s12882-021-02317-x

**Published:** 2021-03-29

**Authors:** Mathijs van Oevelen, Alferso C. Abrahams, Willem Jan W. Bos, Mariëlle H. Emmelot-Vonk, Simon P. Mooijaart, Merel van Diepen, Brigit C. van Jaarsveld, Anita van Eck van der Sluijs, Carlijn G. N. Voorend, Marjolijn van Buren, on behalf of the DIALOGICA study group

**Affiliations:** 1 Department of Internal Medicine, Leiden University Medical Center, Albinusdreef 2, 2333 ZA Leiden, The Netherlands; 2 Department of Nephrology and Hypertension, University Medical Center Utrecht, Utrecht, The Netherlands; 3 Department of Internal Medicine, St Antonius Hospital, Nieuwegein, The Netherlands; 4 Department of Geriatrics, University Medical Center Utrecht, Utrecht, The Netherlands; 5 Department of Gerontology and Geriatrics, Leiden University Medical Center, Leiden, The Netherlands; 6 Department of Clinical Epidemiology, Leiden University Medical Center, Leiden, The Netherlands; 7 Department of Nephrology, Amsterdam Cardiovascular Sciences, Amsterdam University Medical Center, Amsterdam, The Netherlands; 8 Department of Nephrology, Haga Hospital, The Hague, The Netherlands

**Correction to: BMC Nephrol 22, 39 (2021)**

**https://doi.org/10.1186/s12882-021-02235-y**

Following publication of the original article [1], the authors informed us that would like to update the **Acknowledgements** section by giving credit to someone who played a key role in the initial design of the study as follows:

We would like to thank dr. G.A. de Wit, associate professor Health Technology Assessment at the University Medical Center Utrecht, for her work on the design of DIALOGICA.
