# Supplementary material for: DIALysis or not: Outcomes in older kidney patients with GerIatriC Assessment (DIALOGICA): rationale and design
Source: BMC Nephrol. 2021 Jan 23;22:39. doi: 10.1186/s12882-021-02235-y (PMC7825220; doi:10.1186/s12882-021-02235-y)
Supplement: Supplementary file 1 — Additional file 1: Table S1. The Dialysis Symptom Index. [file 12882_2021_2235_MOESM1_ESM.docx]

| **Table S1. The Dialysis Symptom Index^30^** | |
| --- | --- |
| 1. Constipation | 16. Chest pain |
| 2. Nausea | 17. Headache |
| 3. Vomiting | 18. Muscle soreness |
| 4. Diarrhoea | 19. Difficulty concentrating |
| 5. Decreased appetite | 20. Dry skin |
| 6. Muscle cramps | 21. Itching |
| 7. Swelling in legs | 22. Worrying |
| 8. Shortness of breath | 23. Feeling nervous |
| 9. Light-headedness or dizziness | 24. Trouble falling asleep |
| 10. Restless legs or difficulty keeping legs still | 25. Trouble staying asleep |
| 11. Numbness or tingling in feet | 26. Feeling irritable |
| 12. Feeling tired or lack of energy | 27. Feeling sad |
| 13. Cough | 28. Feeling anxious |
| 14. Dry mouth | 29. Decreased interest in sex |
| 15. Bone or joint pain | 30. Difficulty becoming sexually aroused |
