# Supplementary material for: DIALysis or not: Outcomes in older kidney patients with GerIatriC Assessment (DIALOGICA): rationale and design
Source: BMC Nephrol. 2021 Jan 23;22:39. doi: 10.1186/s12882-021-02235-y (PMC7825220; doi:10.1186/s12882-021-02235-y)
Supplement: Supplementary file 2 — Additional file 2: Table S2. Categories for mortality, using ERA-EDTA codes, based on the United Kingdom Renal Registry. [file 12882_2021_2235_MOESM2_ESM.docx]

| **Table S2. Categories for mortality, using ERA-EDTA codes, based on the United Kingdom Renal Registry^44, 45^** |
| --- |
| Cardiac disease (including myocardial ischaemia and infarction, cardiac arrest/sudden death, hypertensive cardiac failure, fluid overload/pulmonary oedema and other causes of cardiac failure); |
| Cerebrovascular accident; |
| Infection (including all causes of peritonitis, septicaemia, tuberculosis, pulmonary infections, generalised viral infection, infections elsewhere except viral hepatitis) |
| Treatment withdrawal (including patients refusal of treatment, withdrawal due to medical reasons, and cessation for any other reason); |
| Malignancy; |
| Other causes; |
| Uncertain aetiology / undetermined causes of death |
