# Supplementary material for: DIALysis or not: Outcomes in older kidney patients with GerIatriC Assessment (DIALOGICA): rationale and design
Source: BMC Nephrol. 2021 Jan 23;22:39. doi: 10.1186/s12882-021-02235-y (PMC7825220; doi:10.1186/s12882-021-02235-y)
Supplement: Supplementary file 3 — Additional file 3: Table S3. Categories for hospitalisation, using ICD-10 codes. [file 12882_2021_2235_MOESM3_ESM.docx]

| **Table S3. Categories for hospitalisation, using ICD-10 codes^46^** |
| --- |
| Cardiac (including myocardial ischaemia/infarction, cardiac arrest/arrhythmia, cardiac failure, fluid overload/pulmonary oedema, haemorrhagic pericarditis); |
| Vascular disease (including pulmonary embolus, stroke, cerebrovascular haemorrhage, ruptured vascular aneurysm, mesenteric infarction, peripheral arterial disease); |
| Infection, non-dialysis related (including bacteraemia/sepsis, cardiac infection, HIV, osteomyelitis, respiratory infection, urinary tract infection); |
| Dialysis related (including dialysis access infection, peritonitis, PD catheter leakage/exchange/removal, fistula operation, renal fluid overload, bleeding); |
| Malignancy; |
| Bleeding, non-dialysis related (including intracranial bleeding, gastro-intestinal bleeding, other causes of bleeding); |
| Other causes. |
